# Advanced Regenerative Medicines for Rare Diseases: A Review of Industry Sponsors Investment Motivations

**DOI:** 10.1007/s43441-024-00690-x

**Published:** 2024-09-10

**Authors:** Ubaka Ogbogu, Anja Nel

**Affiliations:** 1 Faculty of Law, University of Alberta, Edmonton, Canada; 2 Alexander Holburn, Vancouver, B.C. Canada

**Keywords:** Advanced regenerative medicines, Rare diseases, Orphan drugs, Drug regulation

## Abstract

Despite regulatory changes designed to stimulate investment in therapies for rare diseases, many of these conditions lack government-approved treatments. Advanced regenerative medicines, which are therapies and clinical interventions aimed at healing or replacing damaged or defective human cells, tissues, and organs, offer great promise for addressing many rare diseases. A major challenge facing advanced regenerative medicines for rare diseases is securing financial support to assist in bringing a therapy to market. This paper describes the factors cited by pharmaceutical industry players globally for sponsoring the development of advanced regenerative medicines for rare diseases. The paper examines the motivations of 53 sponsors that meet the latter criteria. The motivations behind investments were broadly similar amongst sponsors and map closely onto regulatory requirements for clinical development and marketing authorization of advanced therapeutic products, including the presence of accelerated or attenuated pathways for regulatory approval, use for indications with high unmet medical needs, and/or that have advantages over existing therapies, and robust preclinical data. Other factors include availability of investment incentives and opportunities for off-label use in the post-approval stages.

## Introduction

A rare disease is a disease or medical condition that affects a small number of individuals in a given population unit, such as a country. Globally, there are an estimated 6,000 to 8,000 rare diseases [1]. While rare in proportion to a given population, the collective number of persons impacted by rare diseases is estimated to be between 3.5 and 5.9% of the global population, corresponding to 263 to 446 million people [2].

Although what is considered a rare disease varies by jurisdiction, rare diseases are chiefly defined by low prevalence. In Canada and the European Union (“EU”), a disease is considered rare if it affects no more than one in 2000 people [3–4]. The United States Food and Drug Administration (“USFDA”) defines a condition as rare if it affects fewer than 200,000 individuals in the United States (“U.S.”) [5]. Japan, Australia, and South Korea define rare diseases similarly to include life-threatening or seriously debilitating conditions that impact only a small segment of that country’s population, or that are unlikely to be financially viable for the sponsor to market [6–8]. The latter considerations are also present in what constitute a rare disease in the U.S. and the EU.

Although there is no unified international framework, countries have adopted regulatory approaches known as “orphan drug” frameworks to categorize and incentivize research and development (“R&D”) pertaining to rare disease therapies. For example, the United States Orphan Drug Act of 1983 provides a range of financial and regulatory incentives to pharmaceutical companies to foster the development of therapies for rare diseases, such as federal grants, tax credits, and regulatory fee waivers [9]. Similarly, in Canada, companies developing rare disease therapies are eligible for the Scientific Research and Experimental Development program tax incentive, which includes refundable tax credits, indefinite carry forward of R&D expenditures, and tax deductions for capital equipment for R&D, among other incentives [10]. Other jurisdictions offer incentives that include access to accelerated or expedited health products approval programs, fee rebates, data/patent protection, and a guaranteed period of market exclusivity for approved orphan drugs [11].

Despite improvements in the ability to detect, diagnose, and discover rare diseases, nearly 95% of these conditions lack approved treatment options [12]. The lack of approved treatments extends to advanced regenerative medicines (“ARM”), which are novel and innovative therapies and clinical interventions aimed at healing or replacing damaged or defective human cells, tissues, and organs, and which seek mainly to address incurable conditions. Examples of ARM include stem cell therapies, gene therapy, and tissue-engineered therapies. Although ARM offer great promise for addressing many rare diseases, much like other rare disease therapies, those that target rare diseases face challenges with securing financial support for research and product development given the inherently small patient populations [13]. These challenges are likely compounded for ARM that are prescribed as one-time treatments, such as some gene therapies. While financial support from governments and the public sector can help, the challenge remains due to other exacerbating factors such as the complexity of ARM, exorbitant costs of drug development, and a resulting lack of certainty regarding regulatory approval and return on investment [14]. The majority of companies focused on ARM for rare diseases are small and medium-sized enterprises, and have fewer resources for and experience in operating this costly endeavour [].

Regulatory developments that clarify, facilitate, and, in some cases, expedite the process for regulatory review and approval of emerging therapies can alleviate the financial and R&D challenges. In the U.S., for example, sponsors of ARM for rare diseases can apply to have their products designated as a “regenerative medicine advanced therapy” (“RMAT”). The designation, which was introduced by legislation in 2016, applies to cellular therapies (allogeneic and autologous), tissue engineering products, human cell and tissue products, human gene therapies, and combinations thereof. Developers of RMAT-designated products that address conditions with high unmet medical need can apply for expedited regulatory and marketing approval, alongside other incentives. Other jurisdictions, including Canada and the EU, have made regulatory changes that provide similar incentives (we elaborate on these regulatory incentives in Sects. Orphan Drug Regulation and Expedited Regulatory Review of the paper).

While it is not yet clear if there is a correlation between products receiving a special designation, such as RMAT, and being approved in an accelerated manner, several ARM for the treatment of rare diseases have been approved in the U.S. since the introduction of the designation [16, 17]. However, whether these regulatory initiatives are having an impact on R&D investment motivations is unclear. Indeed, little remains known about the sponsors of ARM for rare diseases and especially about their motivations for undertaking to sponsor products in this category. Knowledge regarding sponsors (i.e., investors who commit capital to ARM with the expectation of receiving financial returns) and their motivations is crucial to understanding the factors that attract investment in R&D for rare disease therapies, as well as possible reasons as to why sponsors choose to navigate the terrain despite significant challenges. It will also provide vital insights on whether and to what extent regulatory frameworks and developments serve to generate interest in and investment in the area, or conversely, act as a barrier to investment. It is important to note that there are other factors that drive investment in R&D for rare disease therapies, such as advocacy by patients, caregivers, and other affected groups [18–20]. However, discussion of these factors is beyond the scope of this paper.

## Aims and Methods

This paper provides a review of factors that motivate investment in ARM for rare diseases. The term “sponsor” as used in this paper refers to corporations making financial investments to bring ARM for treating rare diseases to market. The paper focuses on who the sponsors are and the considerations motivating their investments in ARM for rare diseases, as gleaned from publicly available information, including information generated by the sponsors themselves.

The review was conducted by one of the authors (AN) through a textual analysis that sought to identify themes in the included materials, as they relate to the factors that motivate sponsors to invest in rare disease ARM. The lead author (UO) carried out a second analysis to validate the themes identified in the primary review and analysis. In Sect. Overview of Investment Considerations of the paper, we provide an overview of the factors identified in our analysis but elaborate only on the factors that represent the most common themes (i.e., the most frequently cited themes) arising from the analysis.

Sponsors included in the review were sourced from a global listing of sponsors of ARM compiled by the Regenerative Medicine Catalyst Project, a life sciences focused consortium based in Australia, regulatory databases of approved therapies, and governmental clinical trial websites [16, 21, 22]. Fifty-three (53) sponsors met our research criteria, defined as those sponsoring ARM for rare diseases approved for clinical use or in late-stage (Phase 2/3) clinical trials. There are two reasons for limiting the review to sponsors who met the above criteria. The first is to capture sponsors who have maintained or are likely to maintain sustained investment and interest in rare disease therapies, and the second is to limit the scope of the review to an analyzable sample.

Our review encompassed sponsor websites, publicly available press releases, and financial documents filed with the Securities Exchange Commission (“SEC”). The paper relies heavily on the SEC filings due to the level of detail in them and their reliability, given that the sponsors themselves made them available to the SEC. In several cases, the SEC filings included detailed agreements between sponsors and academic institutions, involving the development of ARM for rare diseases. The period covered in the review is between January 2020 and December 2022.

Prior to presenting our findings, it is important to elaborate on some key regulatory and investment matters alluded to briefly in this section. Our goal in doing so is to provide important context for understanding the findings and analysis that follow. These matters include orphan drug regulation, regulatory review for drugs and advanced therapeutic products, and investment challenges in the context of bringing rare disease therapies to market. We limit our discussion of these matters to the Canadian, U.S., and EU contexts, as they are largely similar, and because a full discussion of other jurisdictions is beyond the scope of this paper. While there are other factors that influence investment decisions, such as drug pricing, the likelihood of reimbursement from public or private payers, and the costs and challenges associated with developing ‘companion diagnostics’ (medical devices needed for the safe and effective use of a rare disease ARM), we do not elaborate on these matters because our review did not reveal that sponsors consider them to be investment considerations. It is important to state that we are not implying that these factors do not impact investment considerations. Rather, we have not discussed them as doing so is not likely to provide any useful context for the findings presented in this paper. We also do not address factors that motivate or increase investment in regenerative medicine more broadly, such as targeted research funding initiatives, regulatory harmonization, and global cooperation programs that foster strategic collaborations/partnerships between academic institutions, industry players, government agencies, and non-governmental organizations. These topics, though important, are not the focus of this paper, and some have already been addressed in the literature [23].

## Orphan Drug Regulation

Various jurisdictions have enacted orphan drug regulatory policies that aim to lower the cost of drug development and incentivize investment in the rare disease space [24]. These policies establish criteria for designating a drug as an orphan drug (the legal criteria for orphan drug designation in the U.S. and the EU are reproduced in Box A). Canada has not established a formal orphan drug framework or designation. In the U.S., the USFDA, via the Office of Orphan Products Development, grants orphan drug designation where a drug or therapy targets a rare disease or condition [25]. This designation affords a number of incentives to sponsors who develop orphan drugs. These incentives include a guarantee of a seven-year market exclusivity upon regulatory approval of an orphan drug, a guarantee that the USFDA will not approve the same drug for the same orphan indication during the exclusivity period, tax credits for certain qualified clinical testing expenses, and a waiver of the Prescription Drug User Fee Act (“PDUFA”) fee, which is required when sponsors submit a drug for regulatory approval [26]. In eligible cases, the small business waiver can be granted to reduce or remove PDUFA fees. In 2022, the PDUFA application fee exceeded three million dollars where clinical data was required [26]. This translates into cost-savings, which may be attractive to small and medium-sized enterprises operating in this space. Furthermore, sponsors who develop drugs targeting rare pediatric diseases are eligible to obtain “priority review vouchers” from the USFDA. The vouchers can be redeemed to receive a priority review of a future drug, or sold to other developers [27]. Altogether, these incentives have encouraged a steady increase in development in this space with the USFDA receiving over 750 orphan drug designation requests in 2020 (compared to 534 in 2019) [28]. Sponsors included in our review, such as BioMarin, have pursued market exclusivity by strategically developing orphan drug product candidates [29]. In the EU, the European Medicines Agency (“EMA”) provides a ten-year market exclusivity guarantee to developers of orphan-designated medicines which protects against the approval and marketing of a similar product for the same therapeutic indication within the exclusivity period [30, 31]. Other incentives available in the EU include scientific advice specific to orphan medicines, and certain fee reductions [32].

**Box A:** Legal Criteria for Orphan Drug Designation in the U.S. and the EU

**European Union** A medicinal product shall be designated as an orphan medicinal product if its sponsor can establish that it is intended for the diagnosis, prevention or treatment of a life-threatening or chronically debilitating condition affecting not more than five in 10 thousand persons in the Community when the application is made, or that it is intended for the diagnosis, prevention or treatment of a life-threatening, seriously debilitating or serious and chronic condition in the Community and that without incentives it is unlikely that the marketing of the medicinal product in the Community would generate sufficient return to justify the necessary investment, and that there exists no satisfactory method of diagnosis, prevention or treatment of the condition in question that has been authorised in the Community or, if such method exists, that the medicinal product will be of significant benefit to those affected by that condition.	
**United States** The manufacturer or the sponsor of a drug may request the Secretary to designate the drug as a drug for a rare disease or condition…the term “rare disease or condition’” means any disease or condition which affects less than 200,000 persons in the United States, or affects more than 200,000 in the United States and for which there is no reasonable expectation that the cost of developing and making available in the United States a drug for such disease or condition will recovered from sales in the United States of such drug.	

## Expedited Regulatory Review

Sponsors of rare disease therapies may also benefit from expedited or accelerated regulatory pathways, which generally shorten the length of time that it takes to obtain regulatory approval. While these expedited approval pathways are not specific to rare disease therapies, they can serve to motivate investment in such therapies. For example, jCyte, one of the sponsors in our review, identified the availability of expedited regulatory pathways as a key consideration motivating the development of jCell, an allogeneic cell therapy for the treatment of retinitis pigmentosa, a rare eye disease, that results in vision loss [33].

In the U.S., sponsors may apply for expedited development programs for a regenerative medicine therapy candidate where it treats, modifies, reverses, or cures serious conditions [34]. The expedited programs include fast track designation, breakthrough therapy designation for drugs, RMAT designation, accelerated approval for biologics, and priority review designation. These programs offer additional benefits such as quicker regulatory review timelines, guidance from the USFDA to assist with clinical trials or approval submissions, and other assistance, such as efficient drug development guidance to expedite review [35]. Sponsors can also apply for accelerated approval for health products that meet the criteria specified in the Code of Federal Regulations, which applies to “certain biological products that have been studied for their safety and effectiveness in treating serious or life-threatening illnesses and that provide meaningful therapeutic benefit to patients over existing treatments” [35, § 601.40].

Sponsors of regenerative therapies may apply for RMAT designation if the product candidate is intended to treat, modify, reverse, or cure a serious condition and has the potential to address an unmet medical need relating to that condition based on preliminary clinical evidence [34]. Between December 2016 and September 2022, the USFDA received 203 requests for RMAT designation, granting RMAT status to 79 therapies, denying 111 applications, while 9 applications were withdrawn [36]. It is worth noting RMAT designation does not change statutory requirements for marketing approval. The RMAT designation and accelerated approval, in the United States, is correlated with the EU priority medicine designation (“PRIME”) and conditional marketing authorisation. Both EU programs are available to sponsors to assist in accelerated development of therapies for conditions with high unmet need [37, 38]. PRIME designation offers sponsors early and ongoing interactions with EMA to assist with and accelerate the development of a therapeutic product [38]. To be eligible for PRIME designation, the product candidate must demonstrate “a potential benefit [to] patients with unmet medical needs based on early clinical data” [38].

Lastly, in Canada, sponsors may be eligible to apply for accelerated review pathways where the therapy is designed to treat a serious, life-threatening, or severely debilitating condition [39]. These accelerated review pathways reduce Health Canada’s review target of 300 days for a New Drug Submission and enable expedited access to treatments [40].

## Challenges Associated with Bringing Orphan Drugs to Market

Although the introduction of orphan drug frameworks and expedited regulatory pathways have encouraged sponsorship activity in the rare disease space, sponsors that choose to invest in the development or commercialization of these therapies still face numerous significant challenges. Regulatory approval requires comprehensive and scientifically sound data collection involving exorbitant financial and time costs, all of which can be exacerbated by delays in regulatory approval. Financial costs pertain to the monetary resources expended on activities like research, clinical trials, and documentation. Time costs refer to the duration involved in the data collection process and subsequent regulatory approval, both of which can escalate if there are delays in the approval timeline. Identifying eligible patient populations for treatment is also challenging due to a multitude of factors, including limited patient populations, geographical dispersion of patients (giving rise to logistical difficulties in coordinating and conducting clinical trials), heterogeneity of disease presentation and lack of a confirmed diagnosis (both of which can complicate inclusion/exclusion criteria), the lack of comprehensive natural history data for many rare diseases, ethical considerations relating to placement of a small patient population in treatment versus placebo control arms of clinical trials (thereby complicating trial design), lack of regulatory harmonization across jurisdictions, and lack of validated outcome measures rendering it difficult to compare results across trials or to accurately assess treatment effectiveness [41–43]. In particular, differing regulatory processes and guidelines for selecting suitable clinical trial endpoints and appropriate comparators across jurisdictions can delay or impede a sponsor’s bid for access to expedited programs and/or regulatory approval, and may increase administrative burden and R&D costs, especially for small companies or for treatments with limited market potential [43, 44].

Due to the small nature of rare disease patient populations, sponsors will face challenges in ensuring sufficient enrollment of clinical trial participants and often compete with other players in the space to do so. The affordability of treatments may also be a concern, particularly where sponsors rely on governments or insurers providing reimbursements of treatment. For example, bluebird bio withdrew two therapies, Skysona and Zynteglo, from European markets following a lack of agreement among European payers on the value recognition of these therapies [45]. Furthermore, existing commercial frameworks are built for mass-marketed therapies, and ARM for rare diseases may require different manufacturing modalities that add to development costs or frustrate development entirely. Several sponsors in our review indicated that creating in-house manufacturing, and therefore minimizing reliance on third-party manufacturers, is important to the commercialization and scalability of products [46–48]. The reasons why in-house manufacturing may be more advantageous or beneficial than outsourcing (especially from a cost perspective) is not apparent from our review. However, one possible explanation is that given the complexity of ARM therapies, in-house manufacturing allows for more control over the manufacturing process and minimizes quality assurance problems, thus saving costs. Still, in-house manufacturing can be resource-intensive due to high upfront costs of preclinical and manufacturing development and may be out of reach for small-sized developers and new entrants [49].

## Sponsors Profiles

Over half of the 53 sponsors reviewed for this paper are publicly held companies with investment products traded on various financial markets (see Table 1). The majority of them are headquartered in the United States, followed by the EU and the Asia-Pacific region. However, it is common for sponsors to conduct business globally and have several offices in different countries. It is also common for sponsors to have subsidiaries that operate in countries different from where the parent company is domiciled. For example, Kite Pharma, which is focused on cell therapies, is a subsidiary of Gilead Sciences – headquartered in Foster City, California – and has several offices in the U.S., UK, and the Netherlands [50, 51]. Thus, where sponsors are headquartered is not necessarily where they carry on their regenerative medicine operations. The location of operation may depend on the approach to manufacturing – therapies that are manufactured at or close to the point-of-care, such as CAR T-cell therapy, necessitate locating manufacturing sites close to where patients are treated so as to avoid the high cost of shipping patient samples over long distances. It is also safe to conclude that there are active regenerative medicine and/or cell therapy operations in the three main regions identified, i.e., North America, the EU, and the Asia-Pacific region. Perhaps the more interesting and significant observation is that there appears to be no major headquarters or major base of operations in Africa and South America, two regions with a disproportionate number of low- and middle-income economies. This is not to suggest that there are no active operations in these regions, but rather, that the companies in our survey have not set up major administrative or operational bases. While the reasons for this observed disparity were not evident from our review, it is possibly owing to the fact that sponsors developing regenerative medicine products are seeking regulatory and marketing approval in high-income economies and major developed markets, chiefly the U.S. and the EU [21].

**Table:** Table 1 Profile of Included Sponsors, showing headquarters by region, number of employees, type of company, and subsidiaries engaged in ARM for rare diseases

Sponsor name	Headquarters by region	Size (per number of employees)	Type of company	Subsidiaries engaged in ARM for rare diseases
2seventy bio	North America	437	Public	n/a
Abeona Therapeutics	North America	90	Public	n/a
Adaptimmune Therapeutics plc	Europe	494	Public	n/a
AGTC	North America	83	Public	n/a
Amphera	Europe	11	Private	n/a
Astellas Pharma Inc.	Asia-Pacific	15,455	Public	n/a
Atara Biotherapeutics	North America	578	Public	n/a
Autolus Therapeutics plc	Europe	324	Public	n/a
BioGen	Europe, North America	9,610	Public	Nightstar Therapeutics
BioMarin	Europe, North America	3,045	Public	n/a
BioNTech	Europe	3,082	Public	n/a
bluebird bio, Inc.	North America	518	Public	2seventy bio is a tax-free oncology spin off
BrainStorm Cell Therapeutics	North America	43	Public	n/a
Bristol-Myers Squibb Company	North America	32,200	Public	Celgene, Juno Therapeutics
Capricor Therapeutics	North America	48	Public	n/a
CarsGen Therapeutics Holdings Ltd	North America	573	Public	n/a
Castle Creek Biosciences	North America	47	Private	Fibrocell Sciences
CRISPR Therapeutics	Europe	473	Public	Casebia Therapeutics
CSL Behring	North America	25,000	Public	n/a
Dicerna Pharmaceuticals (subsidiary of Novo Nordisk)	North America	300	Private	n/a
Gamida Cell	North America	168	Public	n/a
GenSight Biologics SA	Europe	38	Public	n/a
Gilead Sciences	North America	14,000	Public	Kite Pharma
Holostem Terapie Avanzate	Europe	75	Private	n/a
Iovance Biotherapeutics	North America	319	Public	n/a
jCyte	North America	12	Private	n/a
Krystal Biotech	North America	119	Public	n/a
Legend Biotech	North America	1,000	Public	n/a
Lineage Cell	North America	61	Public	Cell Cure Neuroscience
Lysogene	Europe	21	Public	n/a
MeiraGTx	North America	296	Public	n/a
Neurotech Pharmaceuticals, Inc.	North America	40	Private	n/a
Nippon Shinyaku	Asia-Pacific	2059	Public	NS Pharma
Novartis AG	Europe	104,323	Public	Novartis Gene Therapies
Orca Bio	North America	107	Private	n/a
Orchard Therapeutics plc	Europe	259	Public	n/a
Oxford Biomedica	Europe	940	Public	Oxford Biomedica Solutions
Pfizer	North America	79,000	Public	n/a
PTC Therapeutics	North America	1,177	Public	n/a
Roche	Europe	100,000	Public	Genentech, Spark Therapeutics
Spark Therapeutics (subsidiary of Roche)	North America	700	Private	n/a
Rocket Pharmaceuticals	North America	151	Public	n/a
Roivant Sciences	Europe	1000	Public	Aurvant, Cytovant, Sio Gene Therapies
Sio Gene Therapies	North America	12	Public	n/a
Sangamo Therapeutics	North America	431	Public	n/a
Sarepta Therapeutics	North America	840	Public	n/a
TargaZyme	North America	10	Private	n/a
Tessa Therapeutics	Asia-Pacific	170	Private	n/a
UltraGenyx Pharmaceuticals	North America	1,119	Public	n/a
UniQure	Europe	463	Public	Corlieve Therapeutics
United Therapeutics	North America	965	Public	Northern Therapeutics
VBL Therapeutics	Asia-Pacific	41	Public	VBL, Inc.
Vertex Pharmaceuticals	North America	3,900	Public	n/a

We obtained the approximate size of sponsors in terms of employees from documents filed with the SEC, that noted the number of full-time employees. In the cases of some privately held companies, this information was not readily available and was estimated using LinkedIn profiles or team pages on sponsor websites. Consistent with general trends, most sponsors were SMEs with 500 employees or less, followed by sponsors with 500-5,000, 5,000–15,000, and + 15,000 employees. Some of the largest sponsors analyzed in this review, such as Novartis AG and Roche, are headquartered in Europe yet maintain significant global operations. Mergers and acquisitions are common in the biopharmaceutical industry, so it is important to emphasize that the relative size of sponsors may change considerably. There may also be some discrepancies when a parent company includes the employees of its subsidiaries in its total employee count. For example, the number of individuals employed at Kite Pharma, a Gilead Sciences subsidiary, is included in Gilead Sciences total employee count making it difficult to accurately ascertain the size of Kite Pharma [50].

## Overview of Investment Considerations

Generally, the criteria for investments indicated by sponsors map closely to regulatory requirements, including robust preclinical data, the extent that the biology of the disease is understood, impacts on possible commercial development, and ability to expand to other indications. For example, AGTC has stated that its current focus is to pick rare disease product candidates where there is a straightforward path to the market, sufficient literature information, and preclinical data that closely mimics human genes and provides additional confidence in the underlying science [52]. AGTC has also stated that it will not pursue ultra-orphan drugs, and that the current indications it is pursuing have an estimated 20,000 patient population [52]. Similarly, MeiraGTx is selectively focused on products targeting high unmet medical need and where there is a potential for meaningful clinical benefit [47]. Sponsors may also choose to pursue indications or targets that complement existing product pipelines or ongoing research and development efforts. For example, Sarepta Therapeutics is pursuing gene therapies for both Duchenne Muscular Dystrophy (“DMD”) and Limb-Girdle Muscular Dystrophies (“LGMD”) because the “most severe forms of LGMDs mimic DMD” and the candidate gene therapy for both indications utilizes the same adeno-associated viral vector [53].

Sponsors have various, often overlapping motivations for investing in particular rare disease therapies, such as targeting indications with high unmet medical needs, possible advantages over existing therapies, robust preclinical data and understandings of disease biology, further expansion to broader indications, and straightforward paths to market. These motivations are not discrete, and sponsors tend to have broadly similar investment criteria. For example, BioMarin focuses on indications with significant unmet medical need, well understood biology, and where there are first-to-market or best-in-class opportunities [54].

Some sponsors are also motivated by personal experiences with rare diseases. Lysogene, for example, was founded by Karen Pignet-Aiach to find a cure for MPS III, a devastating disease that impacted her daughter [55].

Below, we discuss in more detail, with examples, three sponsorship or investment rationales that emerged as the most frequently cited factors for the sponsors in our review, including first-to-market opportunities, robust preclinical evidence, and scalability/proof of concept.

### First-to-Market Opportunities

Most, if not all, sponsors mentioned that they actively pursued or aimed to pursue R&D investments that presented the opportunity to be the first therapy to reach the market. Opportunities for reaching this goal abound in the rare disease space because among the 7,000 known rare diseases, over 95% have no treatment [56, 57]. Therapies considered first-to-market include those that are first-in-class, such as AT132, which the sponsor, Astellas Pharma believes is the first gene therapy targeting x-linked myotubular myopathy [58]. Another sub-class that presents first-to-market opportunities is therapies that address high unmet medical need. This agenda echoes orphan drug regulations, which require that candidate therapies target a presently underserved patient population. Unmet medical needs with no approved treatments available present a large market opportunity that can offer significant value to both patients and investors [59]. AGTC, for example, is presently focusing on orphan ophthalmology indications given the extreme value that sight has to patients, and because the small patient population allows for manageable clinical trials while maintaining viable commercial opportunities [60]. However, the company has also stated that if other indications are pursued beyond ophthalmology, their strategy will be focus on those with high unmet medical need and a “strong probability of a streamlined, clinical, regulatory, and commercial pathway” [60].

### Robust Preclinical Evidence

Sponsors also noted that their investment criteria included the availability of promising preclinical data and animal models, and understandings of disease biology and pathways. According to AGTC’s 2021 Annual Report, the company chooses to focus on diseases where “the underlying genetic defect is well characterized” and “can be addressed by approaches amenable” to the company’s technology [60]. AGTC emphasizes that diseases should have existing animal models where “clinical endpoints are objective and accepted by regulatory authorities” [60]. This approach facilitates accumulation of data and accelerates clinical studies and regulatory approval processes [60]. Other considerations include a product’s current stage of development and what type of evidence will be required to demonstrate that it meets regulatory safety and efficacy standards [61].

### Scalability / Proof of Concept

Sponsors typically invest in products or therapies that are scalable to multiple indications and offer “proof of concept” for new or expanded product pipelines and/or building on existing commercial infrastructure. For example, Tessa Therapeutics, a cellular immunotherapy company included in our survey but which dissolved in 2023 due to inability to secure additional investment to support its operations, was focused on developing a cell therapy that targets CD30, which the company described as a “well-validated lymphoma target with homogenous expression in Classical Hodgkin Lymphoma and multiple subsets of non-Hodgkin Lymphomas” [62]. Novartis AG notes that licensed product considerations include those that are complementary to the company’s portfolio and business strategy [61]. Similarly, Castle Creek Biosciences is pursuing therapies that can be maximized to build on infrastructure that will support later product candidates without the need for significant further investments [59]. Sponsors are also likely to pursue rare disease targets that mimic one another, or which utilize the same therapies or therapeutic vectors to help with scalability across technology platforms [53]. Pursuing complementary targets also allows sponsors to minimize R&D costs, maximize infrastructure use, and alleviate the financial challenges associated with developing orphan therapies.

## Limitations

This review is subject to five main limitations. First, the pharmaceutical industry is extremely competitive, and companies are fiercely protective of their intellectual property and insider knowledge. In their SEC filings, companies typically request redaction or omission of documents or information deemed confidential; therefore, publicly available SEC filings may not contain all of a sponsor’s available information. Second, privately held companies are not required to submit information to the SEC. Therefore, our sample does not represent the entirety of sponsors operating in this sector. Third, when a sponsor is a subsidiary of a larger parent company, the information we retrieved may be limited to the parent company. Fourth, this study relies on publicly available information and may not represent the full scope of information available for analysis. Fifth, it is important to note that our selection of sponsors to include in the review was not systematic and may be subject to selection biases. As a result, the findings presented should be interpreted with caution, as they may not represent definitive or exhaustive conclusions regarding sponsors’ investment motivations.

## Conclusion

This review highlights three main considerations that motivate sponsors to invest in the development of ARM for rare diseases, including therapies that are or have the potential to be the first on the market, the presence of robust preclinical evidence to support clinical R&D, and the potential for scalability to new or expanded product lines. The review suggests that regulations and regulatory processes for clinical translation and market authorization are influential in sponsors’ decisions regarding investment in ARM for rare diseases. Sponsors’ investment considerations are not merely incidental to or a by-product of regulatory frameworks. Rather, the connection between both seems mutually reinforcing in the sense that sponsors are reacting to shifts in the regulatory systems that are themselves the result of learnings from the R&D that is occurring in the rare disease space.

## Data Availability

The datasets collected and analyzed for this paper are available in the Open Science Framework (OSF) Repository, https://osf.io/3a2yj/, 10.17605/OSF.IO/3A2YJ.
