# Correction: Advanced Regenerative Medicines for Rare Diseases: A Review of Industry Sponsors Investment Motivations

**DOI:** 10.1007/s43441-024-00699-2

**Published:** 2024-09-16

**Authors:** Ubaka Ogbogu, Anja Nel

**Affiliations:** 1 Faculty of Law, University of Alberta, Edmonton, B.C Canada; 2 Alexander Holburn, Vancouver, B.C Canada

**Therapeutic Innovation & Regulatory Science**

10.1007/s43441-024-00690-x

Due to a typesetting error, the in-text callout for Reference 15 is missing. It should be located at the end of the fourth paragraph in the Introduction section of the article, following Reference 14.

